# Epileptogenesis in Common Parasitic Infections

**DOI:** 10.1007/s11910-022-01187-6

**Published:** 2022-03-25

**Authors:** Rajarshi Mazumder, John K. Lee

**Affiliations:** Department of Neurology, David Geffen School of Medicine, University of California, 710 Westwood Plaza, C109, Los Angeles, CA 90095 USA

**Keywords:** Epileptogenesis, Malaria, Neurocysticercosis, Onchocerciasis, Acquired epilepsy

## Abstract

**Purpose of the review:**

Neurocysticercosis (NCC) has been well recognized as a leading cause of epilepsy. More recently, studies of other parasitic diseases such as cerebral malaria (CM) and onchocerciasis are yielding novel insights into the pathogenesis of parasite-associated epilepsy. We compare the clinical and electrophysiological findings in epilepsy associated with these highly prevalent parasites and discuss the mechanisms involved in epileptogenesis.

**Recent Findings:**

Electrophysiological and imaging biomarkers continue to emerge, and individuals who are at-risk of developing parasite-associated epilepsies are being identified with greater reliability. While both *Taenia solium* and *Plasmodium falciparum* directly affect the brain parenchyma, *Onchocerca volvulus* is not known to invade the central nervous system. Thus, the causal association between *O. volvulus* and epilepsy remains controversial.

**Summary:**

Both NCC and CM have a well-defined acute phase when the parasites directly or indirectly invade the brain parenchyma and lead to local inflammatory changes. This is followed by a chronic phase marked by recurrent seizures. However, these stages of epileptogenic process have not been identified in the case of *O. volvulus*.

## Introduction

Epilepsy is one of the most common neurological disorders globally, affecting approximately 50 million people worldwide [1]. The causes of epilepsy are varied and multifactorial, including genetic and acquired etiologies, such as stroke, brain injury, and neurotoxicity [2]. Of the acquired etiologies, central nervous system (CNS) infections are an important acquired cause of seizures and epilepsy, especially in the low- and middle-income countries. The worldwide pool incidence rate of epilepsy is 61.4 per 100,000-person years (95% CI 50.7–74.4), with a higher incidence rate in low-/middle-income (LMI) countries than in high-income (HI) countries [3]. While there are numerous factors contributing to this disparity including socioeconomic status [4], this may also be due to the higher rates of CNS infections and traumatic brain injury in low-/middle-income countries [5, 6].

Of the infectious etiologies of acquired epilepsy, bacteria, viruses, fungi, and parasites are well-known causes of seizures worldwide. Epilepsy has been long recognized as sequelae of meningitis and encephalitis [7], with a higher incidence in LMI countries [8] than in HI countries. Per Baraff et al*.*, the mean probability of unprovoked seizures after bacterial meningitis was 4.2% [9] in a meta-analysis of 45 studies. The three most common causes of meningitis are meningococcus, pneumococcus, and *Haemophilus influenzae* B (HiB). However, due to factors such as vaccination and access to healthcare, the distribution and proportion of meningitis caused by these bacteria are different between LMI and HI countries. The causative viruses are far more numerous varying widely in geographic distribution, and the most well-known include Herpes simplex virus, Japanese encephalitis, West Nile virus, human herpes virus 6, and human immunodeficiency virus (HIV) [10]. It is believed that the inflammatory response to the viral and bacterial pathogens plays a key role in epileptogenesis [11–13]. Fortunately, many of the viral and bacterial infections of the brain now can be prevented with vaccines and likely reduce the overall burden of infectious epilepsy through primary prevention.

In contrast to most bacterial and viral infection of brain, parasitic infection usually affects the host chronically [14]. This warrants parasitic causes of epilepsy to be considered with special importance. While highly diverse, parasites can be classified into single-celled organism protozoa and multicellular helminths (worms). There is a significant disparity in the incidence of parasitic diseases between LMI and HI countries, and indeed, most burden of helminth infections worldwide occur in LMI countries [15], where 80% of people with epilepsy reside. In sub-Saharan Africa, exposure to parasitic infection has been associated with an increased prevalence of epilepsy and, importantly, co-infection with multiple parasites has been found to increase the risk of epilepsy [16].

Most parasites known to infect the brain have been implicated in causing seizures. Neurocysticercosis (NCC) has been most well recognized as a leading cause of epilepsy, and recent studies of other parasitic diseases including cerebral malaria (CM) and onchocerciasis are yielding novel insight into the pathogenesis of parasite-associated epilepsy. Understanding the mechanism(s) by which parasitic infections may cause epilepsy will help elucidate the pathophysiology involved in acquired etiologies of epilepsy as well. In this review, we evaluate and compare the clinical and electrophysiological findings in epilepsy associated with common and highly prevalent parasites of interest and discuss pathomechanism involved in epileptogenesis.

## Parasite and Epilepsies

### Neurocysticercosis

One of the most frequently studied and recognized parasitic causes of epilepsy is neurocysticercosis (NCC), an infection of the CNS by the larvae of *Taenia solium*. Although infection with NCC does not always lead to seizures, patients who are symptomatic constitute a significant proportion of persons with epilepsy in endemic regions of Latin America and Asia [17, 18]. Given the high seroprevalence of exposure to multiple parasites infections in low-resource regions, establishing a causal relationship between parasite and epilepsy has been controversial [19]. However, treatment of persons with viable parenchymal cysts with anti-helminthic albendazole showed decreased risk of seizure recurrence, supporting the role of NCC in the epileptogenic process [20, 21]. From a public health perspective, epilepsy associated with NCC is especially important given the preventable nature of the disease is exacerbated by the lack of resources (i.e., availability of diagnostic tools, physicians, and both surgical and pharmacological treatment options) [22].

Consensus about the process by which NCC causes seizures involves two complex and intertwined mechanisms of epileptogenesis: *structural* change to the CNS parenchyma [10, 17] caused by the larval cyst and an *inflammatory* reaction to infection [11]. The parasite likely disrupts the blood–brain barrier and enables influx of inflammatory cells into the parenchyma, consistent with the finding that patients with NCC have higher matrix metalloproteinases (a molecule involved in the breakdown of the blood–brain barrier) compared to healthy individuals [23]. The parasite itself undergoes unique distinguishable changes within the brain: a viable cyst, which does not illicit much inflammatory response, followed by a degeneration of the cyst with surrounding brain edema and, subsequent formation of a granuloma that sometimes leads to calcified brain lesions [24]. Each stage of infection might illicit variable inflammatory responses and determine the overall course of NCC-associated epilepsy [25].

There is evidence from electroencephalogram (EEG) and imaging studies to suggest that the NCC-related structural lesions in the brain parenchyma and recurrent inflammation may also lead to hippocampal sclerosis and subsequent mesial temporal lobe epilepsy [26, 27]. However, it is important to note that while dual pathology of NCC-related lesions and hippocampal sclerosis frequently exists, a causal relationship between the two is yet to established [26]. Typical seizure semiology observed in NCC is focal. EEG abnormalities with NCC are commonly focal as well, manifesting as slowing and epileptiform discharges. While there could be a clinico-electrographic correlation with the anatomical location of the cyst-associated lesions [28, 29], the electrophysiological abnormalities are not always concordant with the site of the NCC lesions [30–32]. Follow-up studies of people with NCC found that seizures occur both acutely and in the chronic phase of the infection and about fifty percent of individuals have recurrence of seizures (defined as occurrence of any seizures after one week of index seizure). The persistence of brain lesions on imaging is particularly associated with increased the risk of seizure recurrence [33]. Moreover, younger individuals and persons with a higher burden of cysts are more likely to experience seizures [34]. Unlike the risk of clinical seizures, the electrographic abnormalities do not depend on the burden of the lesion [35, 36]. Interestingly, persons with NCC-associated epilepsy who have electrographic abnormalities on EEG are also more likely to have hippocampal atrophy [37•]. These electroclinical findings reinforce the hypothesis that epileptogenesis in NCC could be mediated through hippocampal sclerosis [38].

### Malaria

Similar to neurocysticercosis, malaria is another common parasitic disease endemic to many low- and middle-income countries in sub-Saharan Africa, several regions of south and southeast Asia, and South America. Caused primarily by five species of *Plasmodium* (*P. vivax, P. ovale, P. malariae, P. knowlesi,* and *P. falciparum*), malaria is a mosquito-borne parasitic disease that can manifest in various organ systems, with cerebral malaria (CM) as one of the most common causes of seizures in Southeast Asia and Africa [39, 40]. Among the five species of *Plasmodium* known to cause disease in human, P. *falciparum* has the greatest propensity to cause the most malignant form of malaria, i.e., cerebral malaria [39]. The life cycle of this parasite and the pathophysiology of malarial disease has been well established in the literature [41], though the exact mechanism of epileptogenesis is still under investigation.

*Plasmodium* does not invade the brain directly, as it remains inside erythrocytes. Parasite-infected red blood cells sequestered in the intravascular space subsequently cause perivascular damage, which is believed to be the primary step in ictogenesis [42, 43]. This is followed by loss of perfusion to the parenchyma leading to localized ischemia of the brain tissue. This structural change to neurovasculature and its subsequent effect on the brain parenchyma is believed to induce an inflammatory reaction [44], mirroring the mechanisms of epileptogenesis hypothesized in neurocysticercosis. In addition, there are other mechanisms of seizure generation in malaria, though these are often sequelae of non-neurological systemic effects of malaria such as hypoglycemia, severe metabolic acidosis, and shock [45].

Cerebral malaria, a complicated form of malaria, is clinically defined as unarousable coma following the correction of hypoglycemia, detection of *Plasmodium* parasite in the peripheral blood, and absence of any other causes of encephalopathy [46]. Clinically, seizures are commonly seen during the acute phase of both cerebral and uncomplicated form of malaria [47]. About 10% of survivors with CM develop epilepsy within two years [47]. High fever and acute seizures during CM increase the risk of subsequent malaria associated epilepsy [47]. The semiology of seizures associated with CM is typically focal with or without secondary generalization [10, 48, 49]. Status epilepticus also occurs frequently and may contribute to epileptogenesis [50].

In the attempt to further understand CM-associated epilepsy, EEG has been a powerful tool in investigation, treatment, and prognosis [51, 52]. EEG findings during hospitalization predicted morbidity and mortality among of children with malaria [52]. Acutely, electrographic features found on EEG review include focal epileptiform discharges, most commonly in the parieto-temporal regions [50]. A prospective follow-up of 70 children with CM found that while there is no statistically significant difference in the EEG on visual inspection between persons with and without epilepsy, quantitative spectral analysis showed a higher gamma-delta ratio in children with epilepsy who survive CM [53]. This suggests that the presence of gamma activity might serve as a potential biomarker in epileptogenesis in CM, similar to other epilepsies [54].

More than 80% of individuals with CM-associated epilepsy continue to have seizures despite treatment with available epileptic drugs [47]. As EEG and imaging biomarkers continue to emerge and at-risk individuals are identified with greater reliability, strategies to prevent CM-associated epilepsy must be prioritized.

### Onchocerciasis

More recently, epilepsy due to exposure to the arthropod-borne nematode *Onchocerca volvulus* has gained attention [55]. Unlike *T. solium* and *Plasmodium* which invade the brain parenchyma, *Onchocerca volvulus* is not known to directly enter the CNS [56]. Thus, the causal association between *Onchocerca volvulus* and epilepsy has been questioned. However, several epidemiological studies have found an a strong association between the parasite *O. volvulus* and epilepsy [57, 58] and a dose–response relationship was reported between exposure to *O. volvulus* and the risk of developing epilepsy [59, 60••]. Several researchers have proposed an immune-mediated hypothesis in the pathogenesis of epilepsy associated with *O. volvulus* [61, 62]. This hypothesis is supported by the recent discovery of antibodies against leiomodin-1 and DJ-1 in nodding syndrome, an endemic form of epilepsy strongly associated with *O. volvulus* infection [63]. The theorized pathogenesis of epilepsy in such cases involves parasitic infection resulting in the induction of both the innate and adaptive immune responses. This in turn promotes neuroinflammation, as inflammatory molecules cross the blood–brain barrier. Ultimately, this neuroinflammatory response is believed to result in disruption of neural networks and, subsequent, epileptogenesis [62]. There is already precedent for an immune-mediated disease process associated with *O. volvulus,* as in the cases of “river blindness” [64] where immune response to the parasite can cause sclerosing keratitis, chorioretinitis, or optic neuritis. In the case of other recently discovered immune-mediated epilepsies such as NMDAR and anti-LGI1, treatment of the underlying autoimmune disorder often results in improvement of seizures, suggesting that the seizures are driven by the inflammatory process [65–67]. It remains to be seen if anti-inflammatory treatments could alter the course of *O. volvulus*-associated epilepsies.

Multiple seizure types and epilepsy subtypes have been associated with *O. volvulus*, including endemic forms of epilepsy syndromes such as nodding syndrome and Nakalanga syndrome [62, 68–70]. Generalized tonic–clonic seizures are commonly reported semiology associated with *O. volvulus* infection [71]. Subtypes of *O. volvulus*-associated epilepsy include nodding syndrome, an epileptic encephalopathy characterized by atonic seizures with repetitive dorso-ventral head drop, from which the name of the syndrome is derived [72]. The Nakalanga phenotype associated with *O. volvulus* is defined by growth retardation, physical deformities (including severe kyphosis), endocrine dysfunction and generalized tonic–clonic seizures [69]. Electrographic findings of *O. volvulus-*associated epilepsies are also varied, reflecting the clinical heterogeneity. Interictal electroencephalography often revealed bifronto-temporal spike and slow wave epileptiform discharges [68]. Moreover, other findings include frequent generalized slowing of the background activity and, in some focal epileptiform activities [73]. In nodding syndrome, the EEG is very different from what is typically encountered in other forms of epilepsy associated with *O. volvulus.* Interictal EEG is dominated by generalized slow spike-and-wave discharges, as expected with many epileptic encephalopathies [72]. Further details of epileptiform abnormalities associated with nodding syndrome may be found in another comprehensive review [72].

It is unclear whether the clinical heterogeneity of epilepsies associated with *O. volvulus* parasite is due to a misattribution of the cause and a limitation in knowledge regarding other etiological factors. Further studies are needed to evaluate if the parasite *O. volvulus* causes heterogenous forms of epilepsies with variable electroclinical features. Imaging and electroclinical studies with detailed phenotyping of the varied epilepsies associated with *O. volvulus* remain scarce.

Comparing the natural history of the aforementioned parasitic diseases, a stark distinction between *O. volvulus* and the other two parasites, *T. solium* and *P. falciparum*, becomes apparent. In both NCC and CM, there is an acute phase of new-onset seizures as the parasitic invasion of the brain parenchyma leads to local inflammatory changes, which is then followed by a chronic phase of recurrent seizures. In epilepsies associated with *O. volvulus* parasitic infection, however, the latency of developing the disease remains unknown. Further complicating the matter is the lack of any imaging or neuropathological evidence of parasitic invasion of the brain parenchyma. In addition, chronic inflammatory changes involving macrophage clusters have been noted in a minority of cases [74]. An important point to emphasize is that a causal relationship between *O. volvulus* and the development of epilepsy has not yet been definitively established and further research is needed.

### Host Genetics in Parasitic Infection and Epilepsy

Parasites have co-evolved with humans and provided selective pressure on host genetics [75]. Thus, when evaluating the effect of parasites on epileptogenesis, the human–host factors also need to be understood. Several studies have found evidence of host-genetic susceptibility that leads to an increased risk of neuronal injury due to the parasitic infection [76–80, 81•, 82, 83•]. However, the exact mechanism by which host genetics interact with exposure to a parasitic infection and lead to epilepsy remains unknown. Several possibilities could be considered: a) host-genetic predisposition increases the risk of a parasitic infection; b) host genetics affect the immune-response to a parasite leading to epileptogenesis; and c) chronic parasitic infections change the inflammatory milieu and thus lower the seizure threshold in individuals who are genetically susceptible to developing seizures, acting as a “second hit” in the epileptogenic process.

Persons with CM-associated epilepsy commonly have first-degree relatives with epilepsy in their family [76, 77]. A multi-site study involving four African countries found that polymorphism in genes involved in the inflammatory pathways such as IL-10 are associated with acute seizures [78]. The study also found that the association of the genetic polymorphism differed based on the seizure phenotype and the community where the participants were enrolled. Furthermore, host-genetic variants affecting erythrocyte function and tight-junction proteins in the endothelial cells increase the susceptibility to severe malaria infection [79]. Future studies are necessary to evaluate how these varied biological pathways and susceptibility factors ultimately lead to epileptogenesis.

In people with neurocysticercosis, host genetics is also known to play an important role in epileptogenesis. Polymorphisms in Toll-like receptor- 4, which plays an important role in the immune response against NCC within the central nervous system, have been associated with seizure recurrence due to NCC [80, 81•]. Specific genetic variants are more likely to trigger neuroinflammation and subsequent provocation of seizures. One study from Mexico found an association between developing severe NCC and polymorphisms in TRAF-1 (a member of the TNF receptor family) and C5 (a complement component that acts as anaphylatoxins and chemotactic factor). HLA genotyping among families affected with NCC has revealed inherited susceptibility of epilepsy associated with certain HLA haplotypes [82].

Similarly, in *O. volvulus-*associated epilepsy including nodding syndrome, HLA haplotypes have been found to be associated with both protection and susceptibility to the disorder [83•]. Authors have argued that the presence of HLA variants may explain the heterogeneity of the *O. volvulus*-associated epilepsies and why under similar infectious exposure there is such variation in the phenotype, including unaffected family members [83•]. These studies highlight the importance of further research into the role of host genetics in infectious etiologies of acquired epilepsy. Clarifying these pathways may allow for the development of novel anti-epileptogenic treatment mechanisms and achieve primary prevention of epilepsy.

## Conclusion

Infectious etiologies of epilepsy, particularly, parasitic diseases, play a significant role in the burden of neurological disorders worldwide. While primary parasitic infections are preventable, they are hindered by access to resources along with the disparate geographic distribution of the infectious agents. In addition to primary prevention of parasitic infection, future research efforts should focus on understanding how these infectious agents cause neuronal injury that ultimately leads to epilepsy. Electrophysiological and imaging biomarkers continue to emerge, and individuals who are at-risk of developing parasite-associated epilepsies are being identified with greater reliability.
